# pH-Responsive Triblock Copolymeric Micelles Decorated with a Cell-Penetrating Peptide Provide Efficient Doxorubicin Delivery

**DOI:** 10.1186/s11671-016-1755-4

**Published:** 2016-12-05

**Authors:** Khen Eng Ng, Mohd Cairul Iqbal Mohd Amin, Haliza Katas, Muhammad Wahab Amjad, Adeel Masood Butt, Prashant Kesharwani, Arun K. Iyer

**Affiliations:** 1 Centre for Drug Delivery Research, Faculty of Pharmacy, Universiti Kebangsaan Malaysia, Jalan Raja Muda Abdul Aziz, Kuala Lumpur, 50300 Malaysia; 2 Department of Pharmaceutical Sciences, Eugene Applebaum College of Pharmacy and Health Sciences, Wayne State University, 259 Mack Avenue, Detroit, MI 48201 USA

**Keywords:** Polymeric micelle, pH responsive, Triblock copolymer, Drug delivery, Doxorubicin, Polyarginine

## Abstract

**Abstract:**

This study developed novel triblock pH-responsive polymeric micelles (PMs) using cholic acid-polyethyleneimine-poly-l-arginine (CA-PEI-pArg) copolymers. PEI provided pH sensitivity, while the hydrophilic cell-penetrating pArg peptide promoted cellular PM internalization. The copolymers self-assembled into PMs in aqueous solution at above the critical micelle concentration (2.98 × 10^−7^ M) and encapsulated doxorubicin in the core region, with a 34.2% (*w*/*w*) entrapment efficiency. PMs showed pH-dependent swelling, increasing in size by almost sevenfold from pH 7.4 to 5.0. Doxorubicin release was pH-dependent, with about 65% released at pH 5.0, and 32% at pH 7.4. Cellular uptake, assessed by confocal microscopy and flow cytometry, was enhanced by using doxorubicin-loaded CA-PEI-pArg PMs, as compared to free doxorubicin and DOX-loaded CA-PEI PMs. Moreover, 24-h incubation of these PMs with a human breast cancer cell line produced greater cytotoxicity than free doxorubicin. These results indicate that pH-responsive CA-PEI-pArg micelles could provide a versatile delivery system for targeted cancer therapy using hydrophobic drugs.

**Graphical Abstract:**

Graphical of CA-PEI-pArg polymeric micelles as a pH-responsive drug delivery system.

## Background

Chemotherapy is one of the most commonly used approaches to cancer treatment. However, conventional chemotherapeutics cause numerous unfavorable side effects owing to poor water solubility, a lack of selectivity towards cancer cells, and multi-drug resistance [1]. Doxorubicin (DOX) is used to treat breast cancer, ovarian cancer, lymphoma, multiple myeloma, and sarcoma [2]. However, clinical use of DOX is limited by its side effects, which include drug resistance and cardiotoxicity. The P-glycoprotein efflux pump contributes to multi-drug resistance in cancer cells, reducing the efficacy of chemotherapeutic agents [3]. Therefore, higher dose of DOX is needed to achieve the similar chemotherapeutic effect. As a consequence, the high dose of DOX will eventually causes toxicity effects, which is due to non-specific targeting of the drug, such as cardiotoxicity and hematological toxicity. Commercially available formulations of hydrophobic drugs usually contain synthetic solvents to facilitate parenteral administration. However, such solvents can cause certain adverse effects during chemotherapy. For instance, cremophor EL is a vehicle that is used in such formulations and it has been associated with severe hypersensitivity reactions, hyperlipidemia, and erythrocyte aggregation [4, 5].

To reduce the limitations of chemotherapeutics, a versatile drug delivery system is needed to deliver drugs to targeted tumors. In recent years, there have been many advances in drug delivery involving nano-delivery systems. Block copolymer micelles (PMs) have advantages over other types of nanoparticles used in drug delivery, because polymers with unique benefits can be conjugated as blocks to form di- or triblock copolymers. Characteristics important for drug delivery, including the size and stability of PMs, loading capacity, and release kinetics of the loaded drugs, can be modulated by utilizing a range of block copolymers [6]. Therefore, self-assembled PMs can be tailored to optimize their biocompatibility, biodegradability, and blood circulation time. In addition, PMs can be utilized to overcome multi-drug resistance through passive, folate-mediated, pH-sensitive, and thermo-sensitive targeting [1]. pH responsiveness is a particularly appealing targeted drug release strategy, owing to their preferential drug release at slightly acidic pH in the tumor interstitium (6.5–7.0) and lysosomal compartment (around 5.0), in comparison with that of the normal physiological environment (pH 7.4) [7]. Therefore, the mildly acidic pH of the tumor interstitium is considered to be an ideal trigger for drug delivery systems, because it facilitates programmable release of anticancer drugs at tumor sites [8]. Recently, the pH-responsive delivery systems have attracted much attention in the research of controlled drug delivery [9–12].

Polyethyleneimine (PEI) is a synthetic cationic polymer with a high amine density that is employed in gene delivery because it promotes transfection [13]. PEI facilitates drug release from the endosomal compartment and protects it from enzymatic degradation. The cytotoxic effect of low molecular-weight PEI can be reduced by conjugating it with biocompatible polymers or hydrophobic moieties [13, 14]. Despite its common use in gene transfection, PEI can also be used in pH-responsive drug delivery systems. The amine groups in PEI will undergo protonation in acidic environments and the repulsive force between protonated amine groups will then result in a swollen micellar structure. This phenomenon will destabilize the micellar structure and eventually increase the release rates of loaded drugs [15].

The cationic peptide, poly-l-arginine (pArg), is a cell-penetrating peptide that translocates through cell membranes and facilitates cellular uptake. pArg has therefore been utilized in gene therapy [16], protein/vaccine delivery [17], and cancer treatment [18]. Recent studies reported that poly(amino acid)-based nanoparticles could also effectively translocate through cell membranes [19–22].

Cholic acid (CA) is one of the major bile acids in the human body, and it is composed of a steroid unit with three hydroxyl groups and one carboxyl group [23]. Incorporation of a CA moiety into polymers improves their biocompatibility [24]. CA is amphiphilic and organizes into micelles at its critical micelle concentration (CMC). The hydrophobic domains of CA form the inner core segment and act as a potential drug-incorporation site in the PM, which facilitates transportation of the drug cargo across the cell membrane. In addition, the hydrophilic shell of CA enhances PM biocompatibility, preventing opsonization by the mononuclear phagocytic system and prolonging PM circulation in the blood [25].

Herein we introduce a pH-responsive drug delivery system conjugated with cell-penetrating peptide to achieve a stimuli-triggered drug release system, improve the cellular uptake of drugs, and increase the efficacy of anticancer drugs towards cancer cell line. The originality of this work lies on the use of PEI as pH-sensitive moiety and functionalization of micellar surface with cell-penetrating pArg peptide. The integration of pH-responsive strategy with cell-penetrating peptide has been relatively less explored. The combination of pH-sensitive PEI together with cell-penetrating pArg peptide in a drug delivery system is expected to provide greater stability and improve drug efficacy. In addition, the amphiphilic properties of CA formed the hydrophobic core that was used as a site of entrapment for doxorubicin (DOX). The conjugation of CA to PEI was expected to minimize the cytotoxicity effect from PEI and improve the biocompatibility of the drug delivery system. Hydrophilic pArg and amphiphilic CA were conjugated to the PEI main chain through carbodiimide-mediated amide linkage. PEI was used as the backbone of the triblock copolymer and provided pH sensitivity; while the hydrophilic cell-penetrating pArg peptide was used to facilitate the cellular internalization of the PMs.

The aim of this study was to synthesize DOX-loaded pH-responsive CA-PEI-pArg PMs and investigate the physicochemical properties and biological responses of the PMs towards MCF-7 cancer cell line. The in vitro drug release profile and the cellular targeting of DOX-loaded PMs towards MCF-7 cells were determined and evaluated. Later, the in vitro cytotoxicity of the DOX-loaded PMs against MCF-7 cells was also assessed. The CA-PEI-pArg PMs were designed to act as a versatile drug delivery system in order to overcome the limitation of conventional chemotherapeutics.

## Methods

### Materials

CA, PEI (average molecular weight (MW) approximately 1300 Da), PArg (average MW approximately 10,000 Da), N-(3-dimethylaminopropyl)-N′-ethylcarbodiimide (EDC), and triethylamine were purchased from Sigma-Aldrich (St. Louis, MO, USA). DOX was purchased from Calbiochem (Merck KGaA, Darmstadt, Germany). Spectra/Por™ dialysis membranes with MW cut-offs of 1000 or 13,000 g/mol were purchased from Spectrum Labs (Rancho Dominguez, CA, USA). MCF-7 human breast adenocarcinoma cells and WRL-68 human hepatic cells were purchased from the American Type Culture Collection (Manassas, VA, USA). Dulbecco’s modified Eagle’s medium (DMEM), fetal bovine serum (FBS), trypsin-ethylenediaminetetraacetic acid (EDTA), and (3-(4,5-dimethylthiazol-2-yl)-2,5-diphenyltetrazolium bromide) (MTT) were purchased from Life Technologies (Carlsbad, CA, USA).

### Synthesis of the CA-PEI-pArg Copolymer

**Scheme 1:**
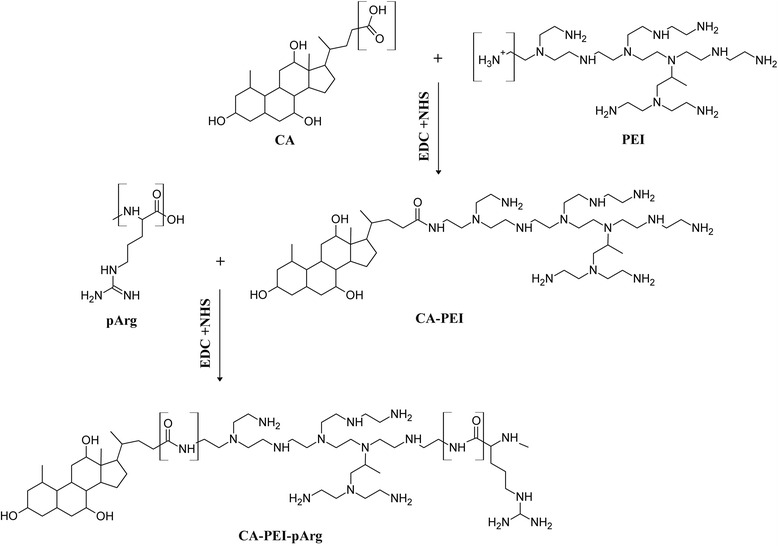
Schematic structure of CA-PEI-pArg triblock copolymer

CA-PEI-pArg was prepared by the two-step reaction method which involves carbodiimide-mediated amidation with EDC and NHS, as described by Li et al. with minor modification [26] (Scheme 1). In Step 1, CA was conjugated with PEI at molar ratios of 1:1, 2:1, or 3:1 to form CA-PEI. To achieve this, CA (47, 94, or 141 mg) in methanol (10 mL) was activated with EDC (44, 88, or 132 mg) and NHS (27, 53, or 80 mg) at room temperature for 6 h. PEI (300 μL) was then added drop-wise into the activated CA solution while stirring. The resulting mixture was stirred at room temperature overnight. The formulation was dialyzed against 500 mL deionized water in a 1000-Da dialysis bag for 48 h and dried by lyophilization.

In step 2, pArg hydrochloride (40 mg) was dissolved in 5 mL methanol, after which 40 μL trimethylamine was added to remove the HCl salt. After 30 min, CA-PEI (40 mg) at the molar ratios indicated above, EDC (40 mg), and NHS (24 mg) in methanol (5 mL) were added drop-wise into the pArg solution. The resulting mixture was stirred at room temperature overnight, dialyzed against 500 mL deionized water in a 1000-Da dialysis bag for 48 h, and dried by lyophilization.

### Characterization of the CA-PEI-pArg Copolymer

CA-PEI-pArg was characterized using a Fourier transform infrared (FTIR) spectrophotometer (Spectrum 100; PerkinElmer, Waltham, MA, USA) and a proton nuclear magnetic resonance (^1^H NMR) spectrophotometer (Bruker Avance III, FT-NMR 600 MHz with cryoprobe; Germany). For FTIR, samples were prepared by compressing the polymers into pellets with potassium bromide. FTIR spectra were recorded over 4000-500 cm^−1^. For ^1^H NMR, chloroform-d (CDCl_3_) was used as the solvent. ^1^H NMR spectra were recorded at 400 MHz.

### Preparation of DOX-Loaded CA-PEI-pArg PMs

DOX-loaded CA-PEI-pArg PMs were prepared by emulsification method, as described by Wahab et al. with minor modification [27]. DOX was prepared by dissolving DOX hydrochloride in chloroform (2 mL) and mixed with triethylamine (2 μL). Excess triethylamine and byproducts were removed by dialysis against 200 mL deionized water in a 1000-Da dialysis bag for 24 h and dried by lyophilization. DOX (5 mg) was dissolved in 2 mL dimethyl sulfoxide, and CA-PEI-PArg copolymers (20 mg) of different molar ratios were dissolved in 5 mL methanol. The DOX solution and CA-PEI-PArg copolymer solution were mixed in a glass vial, kept in the dark for 24 h, and added drop-wise into deionized water (10 mL) under ultrasonic agitation using a Sonifier (Branson Ultrasonics Co., Danbury, CT, USA) at a power of 3 Hz for 10 min. The solvent was evaporated using a rotary evaporator. The DOX-loaded micelle solution was filtered through a syringe membrane filter (0.4 μm) to remove undissolved DOX. The final product was dried by lyophilization.

### Characterization of the CA-PEI-pArg PMs

The CMC of the PMs was determined using dynamic light scattering (DLS) (Zetasizer Nano ZS; Malvern Instruments, Malvern, Worcestershire, UK) at 37 °C with a scattering angle of 90°. The changes in light intensity were recorded for a series of copolymer suspensions at different concentrations. The sample molar concentration was plotted against the mean light intensity. The CMC was the concentration at which a sharp increase in scattering intensity occurred, indicating micelle formation.

Blank and DOX-loaded CA-PEI-pArg PMs (1 mg/mL) were prepared in phosphate-buffered saline (PBS; pH 5.0, 6.0, 6.5, or 7.4). The particle size and zeta potential of the PMs were determined using a Zetasizer Nano ZS (Malvern Instruments) 24 h after preparation. Results were expressed as the average of triplicate measurements.

The morphology of the micelles was analyzed by transmission electron microscopy (TEM; Tecnai™ Spirit, FEI, Eindhoven, Netherlands). Blank and DOX-loaded CA-PEI-pArg PMs were prepared in deionized water (1 mg/mL). A drop of PM suspension containing 0.2% (*w*/*v*) phosphotungstic acid was placed on a copper grid and allowed to dry at room temperature. PM morphology was observed by TEM at an acceleration voltage of 220 kV with various degrees of magnification.

### Drug Loading Content and Entrapment Efficiency

The amount of DOX loaded into the PMs was determined in reference to a standard curve of free DOX. Freeze-dried DOX-loaded CA-PEI-pArg PMs were suspended in deionized water (2 mL), and absorbance was measured at 480 nm using a UV-1601 spectrophotometer (Shimadzu Corp., Kyoto, Japan). Drug loading content (DLC) and entrapment efficiency (EE) were calculated by Eqs. (1) and (2).

1 $$ \mathrm{D}\mathrm{L}\mathrm{C}\ \left(\%\right) = \left(\mathrm{mass}\ \mathrm{of}\ \mathrm{drug}\ \mathrm{encapsulated}\ \mathrm{in}\ \mathrm{micelles}/\mathrm{mass}\ \mathrm{of}\ \mathrm{drug}\hbox{-} \mathrm{loaded}\ \mathrm{micelles}\right) \times 100 $$

2 $$ \mathrm{E}\mathrm{E}\ \left(\%\right) = \left(\mathrm{mass}\ \mathrm{of}\ \mathrm{drug}\ \mathrm{encapsulated}\ \mathrm{in}\ \mathrm{micelles}/\mathrm{mass}\ \mathrm{of}\ \mathrm{drug}\ \mathrm{in}\mathrm{itially}\ \mathrm{added}\right) \times 100 $$

### In Vitro Drug Release from PMs

DOX release studies were carried out using in vitro dialysis at pH 5.0 and pH 7.4. Briefly, freeze-dried DOX-loaded CA-PEI-pArg PMs (equally to 100 μg/mL of DOX) were suspended in deionized water (5 mL). The DOX-loaded PM suspension was placed into 13,000-Da dialysis tubing and dialyzed against 100 mL PBS (pH 5.0 or 7.4) under stirring at 100 rpm at 37 °C. At suitable intervals, 2-mL samples were withdrawn from the release medium and replaced with an equal volume of fresh medium. The released DOX was determined by a UV–VIS spectrophotometer at 480 nm (UV-1601; Shimadzu).

### f2 Similarity Test

The release data were fitted to the f2 equation to investigate the closeness of DOX release profiles from CA-PEI-pArg PMs between pH 7.4 and 5.0. The following equation was used to calculate similarity factors [28].

$$ \mathrm{f}2=50\times \mathrm{Log}\left\{{\left[1+\left(\frac{1}{N}\right){\displaystyle {\sum}_{t=1}n{\left({R}_t-{T}_t\right)}^2}\right]}^{-0.5}\times 100\right\} $$

In the equation, *R*_*t*_ and *T*_*t*_ representing the cumulative percentage released at each of the selected *n* time points under pH 7.4 and 5.0, respectively. f2 was described as a similarity factor. The factor f2 is a logarithmic reciprocal square root transformation of the sum of squared error and it is showing the percentage similarity between the two curves [29].

### Cellular Uptake Study

MCF-7 cells (2.5 × 10^5^ cells/well) were seeded on cover slips placed in six-well plates and allowed for cell attachment by incubating for 24 h. The cells were then treated with free DOX solution, DOX-loaded CA-PEI PMs, or DOX-loaded CA-PEI-pArg PMs (10 μg/mL), and incubated for 1, 4, or 24 h. Di-block CA-PEI PMs were prepared by carbodiimide-mediated amidation and DOX were loaded with emulsification method, as described by Wahab et al. with minor modification [27]. At the designated time points, the medium was discarded and the cells were rinsed twice with cold PBS. The cell nuclei were then stained by Hoechst 33342 for 10 min in darkness. Subsequently, 4% paraformaldehyde was added for 30 min to fix the cells. Finally, the cells were washed twice with cold PBS and mounted on microscope slides using buffered mounting medium. The cells were observed by using confocal laser scanning microscopy (CLSM; TCS SP5, Leica Microsystems, Mannheim, Germany).

The cellular uptake of DOX by MCF-7 cells was analyzed quantitatively by flow cytometry. Owing to the intrinsic fluorescence of DOX, DOX was used as a fluorescent indicator to study the uptake of DOX-loaded PMs. Briefly, cells (1 × 10^6^ cells/well) were seeded in six-well plates, incubated for 24 h, treated with free DOX solution, DOX-loaded CA-PEI PMs, or DOX-loaded CA-PEI-pArg PMs (5 μg/mL), and incubated for 1 or 4 h. Negative control cells were treated with blank micelles. After incubation, the cells were harvested with trypsin-EDTA and resuspended in PBS. The cell suspension was analyzed using a FACS Calibur flow cytometer (BD Biosciences, Franklin Lakes, NJ) and 10,000 events were collected for each sample.

### Cellular and Nuclear Morphology

MCF-7 cells (5.0 × 10^5^ cells/well) were seeded on cover slips placed in six-well plates and incubated for 24 h. The cells were then treated with free DOX solution or DOX-loaded CA-PEI-pArg PMs (5 μg/mL) for 24 h. The untreated cells were used as a negative control group. After incubation, the medium was removed and the cells were washed twice with cold PBS. The cells were then stained by Hoechst 33342 for 10 min in darkness and fixed with 4% paraformaldehyde for 30 min. Finally, the cells were mounted on microscope slides using a buffered mounting medium after washed twice with cold PBS. The cells were observed using CLSM (TCS SP5) and inverted light microscope (Olympus FluoView FV-1000; Olympus Optical Co., Ltd., Tokyo, Japan).

### Subcellular Localization

MCF-7 cells (2.5 × 10^5^ cells/well) were seeded on cover slips placed in six-well plates and incubated for 4 h. The cells were then treated with free DOX solution, DOX-loaded CA-PEI PMs, or DOX-loaded CA-PEI-pArg PMs (5 μg/mL) for 4 h and washed twice with cold PBS. The cells were stained with Lysotracker Green (100 nM) for 30 min and Hoechst 33342 (10 μg/mL) for 10 min in darkness to visualize the lysosomes and nuclei, respectively. The cells were washed twice with cold PBS and fixed with 4% paraformaldehyde for 30 min. The fixed cells were observed by using CLSM (TCS SP5).

### In Vitro Cytotoxicity Study

The cytotoxicity of blank micelles and DOX-loaded micelles were assessed using WRL-68 and MCF-7 cells, which were cultured routinely in DMEM supplemented with 10% FBS and 1% penicillin-streptomycin in a humidified atmosphere of 5% CO_2_ at 37 °C. Cells were seeded in 96-well plates (2 × 10^4^ cells/well for WRL-68 cells and 1 × 10^4^ cells/well for MCF-7 cells) and incubated for 24 h. Blank micelles (3.91–250 μg/mL) were added to WRL-68 cells and MCF-7 cells. Free DOX and DOX-loaded micelles were added to MCF-7 cells (1.562–50 μg/mL). The untreated cells were used as a negative control group. All the cells were incubated for 24 h at 37 °C in a 5% CO_2_/95% air atmosphere. After this 24-h incubation, 20 μL MTT solution (Invitrogen, Carlsbad, CA, USA) was added to each well and the cells were incubated for 4 h. Crystalline formazan was dissolved using acidified isopropyl alcohol (0.04 N HCl). The absorbance of each sample was measured using a microplate reader (Varioskan Flash; Thermo Scientific, Waltham, MA, USA) at 570 nm.

### Statistical Analysis

Values are presented as the mean ± standard error of the mean of triplicate measurements. The statistical significance of differences was tested using two-way analysis of variance. The level of significance was set at *α* = 0.05.

## Results and Discussion

### Synthesis and Characterization of CA-PEI-pArg Copolymer

Synthesis of the pH-sensitive CA-PEI-pArg copolymer was achieved by the two-step reaction process illustrated in Scheme 1. The carboxyl groups of CA and pArg were activated by EDC to form an active O-acylisourea intermediate that was readily displaced by the nucleophilic attack of the amine group of PEI. The primary amine groups of PEI then formed an amide linkage with the carboxyl group of CA. PEI with a MW of 1300 Da was used throughout the study because low-MW PEI could minimize the cytotoxicity of PEI [13]. pArg with a low MW of 10 kDa was used to facilitate renal clearance of pArg after the disintegration of CA-PEI-pArg copolymers in the blood circulation, thus reducing the potential for toxicity [30].

**Fig. 1:**
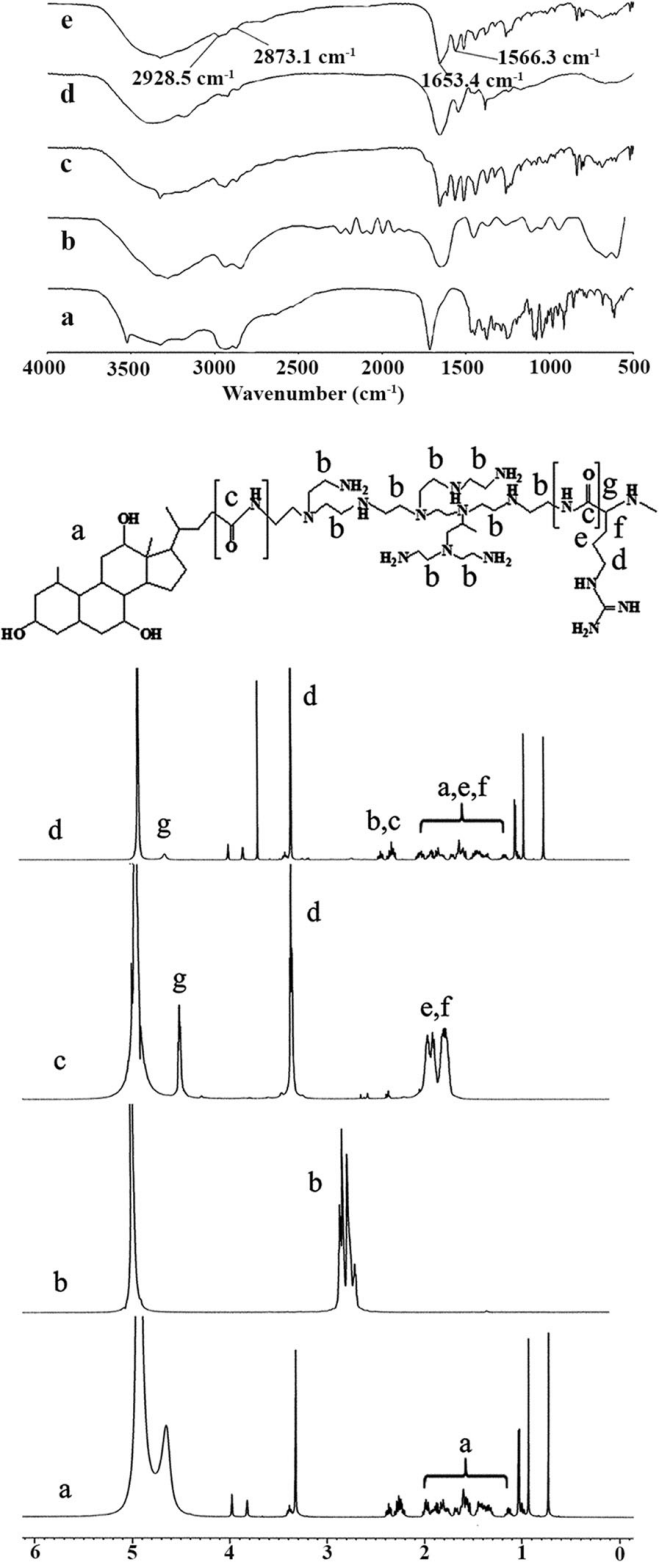
**i** The FTIR spectra of pH-sensitive CA-PEI-pArg copolymer. (*a*) CA, (*b*) PEI, (*c*) CA-PEI, (*d*) pArg, (*e*, *f*, *g*) CA-PEI-pArg. **ii** ^1^H NMR spectrum of (*a*) CA, (*b*) PEI, (*c*) pArg, and (*d*) CA-PEI-pArg

The structure of the CA-PEI-pArg copolymer was characterized by FTIR and ^1^H NMR (Fig. 1). In Fig. 1i (e), FTIR peaks for N–H stretching, C–H stretching, C=O stretching, and N–H bending appeared at wavelengths of 2969, 2867, 1653, and 1564 cm^−1^, respectively, indicating the presence of an amide linkage in the copolymer. FTIR spectra from different sets of CA-PEI-pArg samples showed consistent results, indicating the formation of the amide linkage in all of the formulations. Although the spectra clearly demonstrated the presence of an amide linkage, incorporation of pArg into the CA-PEI copolymer could not be confirmed, because both reactions involved an amide linkage and the spectra were within the same absorbance range.

Formation of the CA-PEI-pArg copolymer was confirmed by ^1^H NMR. As shown in Fig. 1ii (d), proton shifts were detected from 1.0-2.4 ppm, demonstrating the presence of CA in the copolymer. Doublet, triplet, and multiplet peaks were representative of the CA structure. Proton shifts from 3.1-4.0 ppm were characteristic of carbonyl bonds. These results demonstrated the presence of an amide linkage in the CA-PEI-pArg copolymer. In addition, comparison of the ^1^H NMR spectra of the triblock copolymer (Fig. 1ii (d)) with those of the other components (Fig. 1ii (a–c)) showed that distinct chemical shifts attributable to different components were present in the spectrum of the CA-PEI-pArg copolymer. These results confirmed the synthesis of the triblock copolymer.

### Characterization of CA-PEI-pArg PMs

**Fig. 2:**
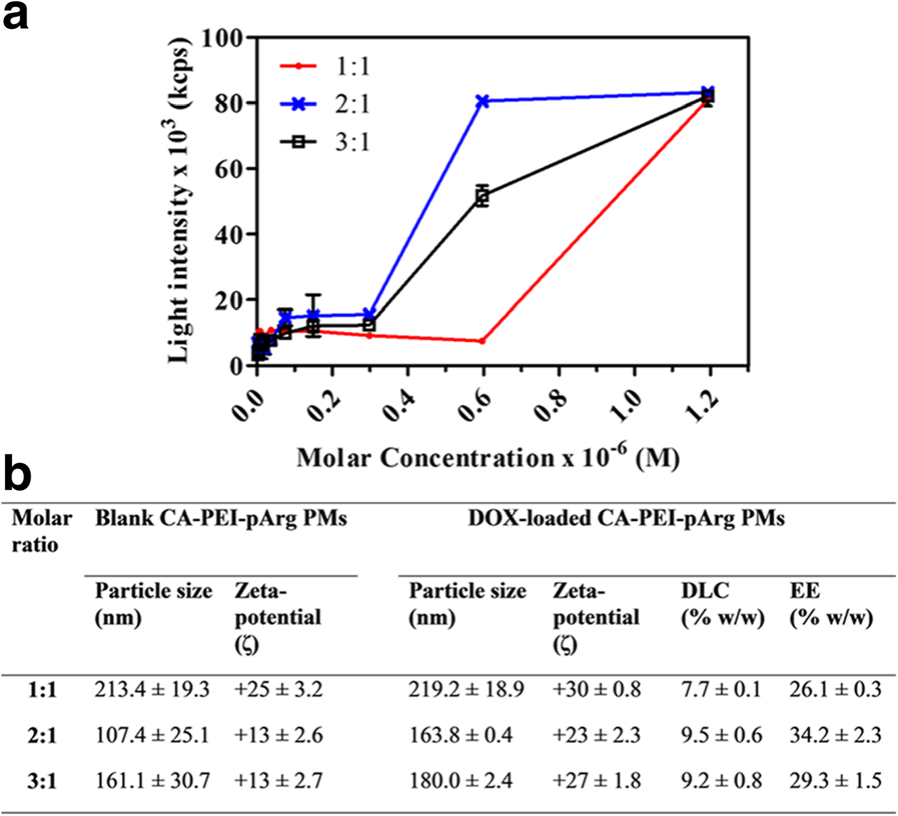
**a** Critical micelle concentrations (CMC) of CA-PEI-pArg PMs. **b** Physicochemical properties of blank and DOX-loaded CA-PEI-pArg PMs (mean ± SEM, *n* = 3)

Block copolymers self-assemble and rearrange to form PMs above their particular CMC. The CMCs of CA-PEI-pArg PMs with different molar ratios are shown in Fig. 2a. An abrupt increase in light intensity was measured via DLS, indicating micelle formation. The CMCs for PMs with CA:PEI molar ratios of 3:1 (2.98 × 10^−7^ M) and 2:1 (2.98 × 10^−7^ M) were lower than that of PMs with a molar ratio of 1:1 (5.96× 10^−7^ M). The ratio of hydrophilic to hydrophobic segments affects the CMC [31]. CA has a hydrophobic steroidal nucleus in its molecular structure, and increased CA substitution may therefore enhance the hydrophobic interactions between the block copolymers in the core region of the micelle, allowing copolymers to stabilize and self-assemble into micellar structures at lower concentrations. Lower CMCs improve micelle stability in dynamic in vivo systems. It is essential that intravenously administered micelles maintain their integrity in blood vessels and it is therefore crucial that clinically used micelles have low CMCs, in order to prevent micelle collapse due to the dilution effect following administration.

The zeta potential of the micelles was determined and recorded (Fig. 2b). Zeta potential can influence particle stability in dispersion due to the electrostatic repulsion between particles with same type of charge. As expected, all the blank and DOX-loaded PMs possessed a positive surface charge due to the high density of amines on the hydrophilic shell. This would prevent agglutination of PMs in the dispersion. DOX-loaded PMs showed a higher zeta potential than blank PMs. This increase in zeta potential reflected the increase in particle size, due to the entrapped DOX. Cellular membranes exhibit anionic surface charges due to the presence of phospholipid bilayer and membrane-bound proteins [32]. Therefore, electrostatic interaction of cationic PMs with cellular membranes could enhance nanoparticle internalization and improve cellular uptake of DOX. However, the zeta potential must be monitored carefully because strongly cationic polymers can be highly cytotoxic [33]. Hence, PMs with CA:PEI molar ratios of 2:1 and 3:1 had an advantage over those with a 1:1 ratio due to their lower zeta potential, which would possibly cause lower toxicity.

DOX encapsulation in the CA-PEI-pArg PMs was evaluated by measuring DLC and EE. Of the tested formulations, PMs with a molar ratio of 2:1 had the highest DLC (9.5% *w*/*w*) and EE (34.2% *w*/*w*). It was hypothesized that hydrophobic DOX would interact with the hydrophobic block copolymers in an aqueous environment and become trapped within the core regions of the micelles. Therefore, PM hydrophobicity could affect its capacity to encapsulate hydrophobic drugs. The PMs with molar ratios of 2:1 and 3:1 had greater degrees of CA substitution than the PMs with a molar ratio of 1:1, conferring a greater DLC. The physicochemical properties of CA-PEI-pArg PMs are summarized in Fig. 2b.

### pH Sensitivity of the PMs

**Fig. 3:**
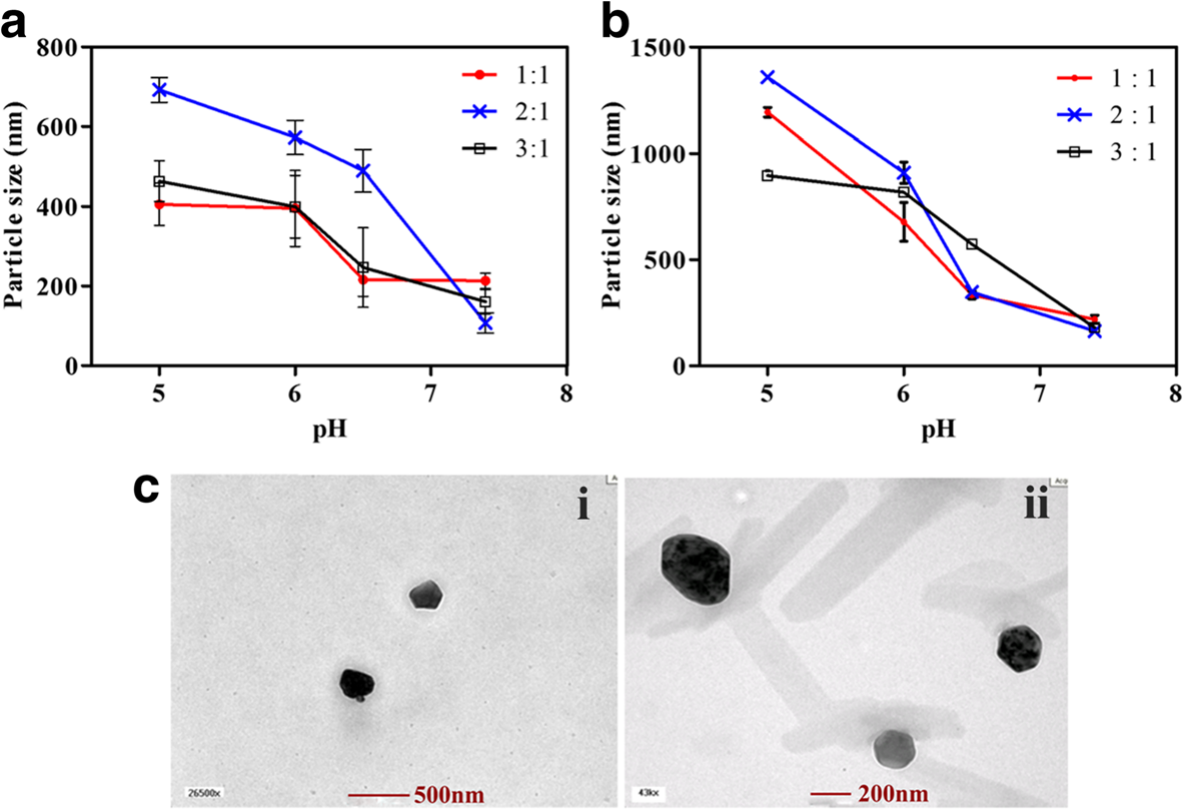
**a** Particle sizes of pH-responsive blank CA-PEI-pArg PMs at pH 5.0, 6.0, 6.5, and 7.4. **b** Particle sizes of pH-responsive DOX-loaded CA-PEI-pArg PMs at pH 5.0, 6.0, 6.5, and 7.4. **c** TEM images of blank and DOX-loaded CA-PEI-pArg PMs with CA:PEI molar ratio 2:1. (*i*) Blank CA-PEI-pArg PMs at pH 7.4 and (*ii*) DOX-loaded CA-PEI-pArg PMs at pH 7.4 (mean ± SEM, *n* = 3)

The particle sizes of the blank and DOX-loaded CA-PEI-pArg PMs were determined at different pH values by DLS. The CA-PEI-pArg triblock copolymers (1 mg/mL) of different CA:PEI molar ratios were suspended in PBS (pH 5.0, 6.0, 6.5, or 7.4) and micellar size was recorded (Fig. 3a, b). At pH 7.4, average sizes of both blank and DOX-loaded PMs with different molar ratios ranged from 107.4 to 219.2 nm. As the pH decreased, the micellar sizes of all tested PMs increased markedly. The particle size of the blank and DOX-loaded PMs with a 2:1 molar ratio increased from 107.4 to 692.2 nm (approximately sevenfold) and from 163.8 to 1357.7 nm (approximately eightfold), respectively, with a decrease in pH from 7.4 to 5.0. In CA-PEI-pArg PMs, the amide linkage of PEI (with a high amine content) between hydrophilic pArg and amphiphilic CA serves as the pH-sensitive moiety. It was hypothesized that ionization of copolymer groups altered the polymer conformation as the pH and ionic composition of the aqueous medium changed [34]. Therefore, the amine groups of PEI became protonated and altered conformation in acidic environments. In addition, protonated amine groups possess a greater charge density, resulting in stronger electrostatic repulsion among the hydrophilic moieties, which leads to a swollen micellar structure and a greater PM size [15, 34]. PMs with a CA:PEI molar ratio of 2:1 showed a greater size increase than those with molar ratios of 1:1 or 3:1. In the CA-PEI-pArg copolymer, increasing the degree of CA substitution directly affected the amount of free amine groups in the copolymers. The PMs with the 3:1 molar ratio had the fewest free amine groups; electrostatic repulsion between the hydrophilic block copolymers was therefore weaker than that observed in the PMs with other molar ratios. In addition, the increase in CA substitution also increased the hydrophobicity of the copolymer, resulting in stronger hydrophobic interactions within the core region and stabilization of the PM structure [35]. Surprisingly, PMs with a 1:1 molar ratio showed a smaller change in size than PMs with a 2:1 molar ratio, perhaps reflecting the high hydrophilicity conferred by pArg (there are more primary amine groups on PEI, which are readily conjugated by pArg). The increased hydrophilic to hydrophobic ratio could stabilize the PM structure due to hydrogen bonding between lone-pair electrons in amines and protons in water molecules.

The morphology of blank and DOX-loaded CA-PEI-pArg PMs was observed by TEM (Fig. 3c). The micelles exhibited a uniform spherical morphology at all tested molar ratios. The micellar size determined by TEM was slightly smaller than that measured by DLS, because the sizes obtained from the DLS measurements represent the hydrodynamic diameter in a micellar suspension whereas the TEM sample preparation involved a drying process that slightly shrank the micelles [36, 37].

**Fig. 4:**
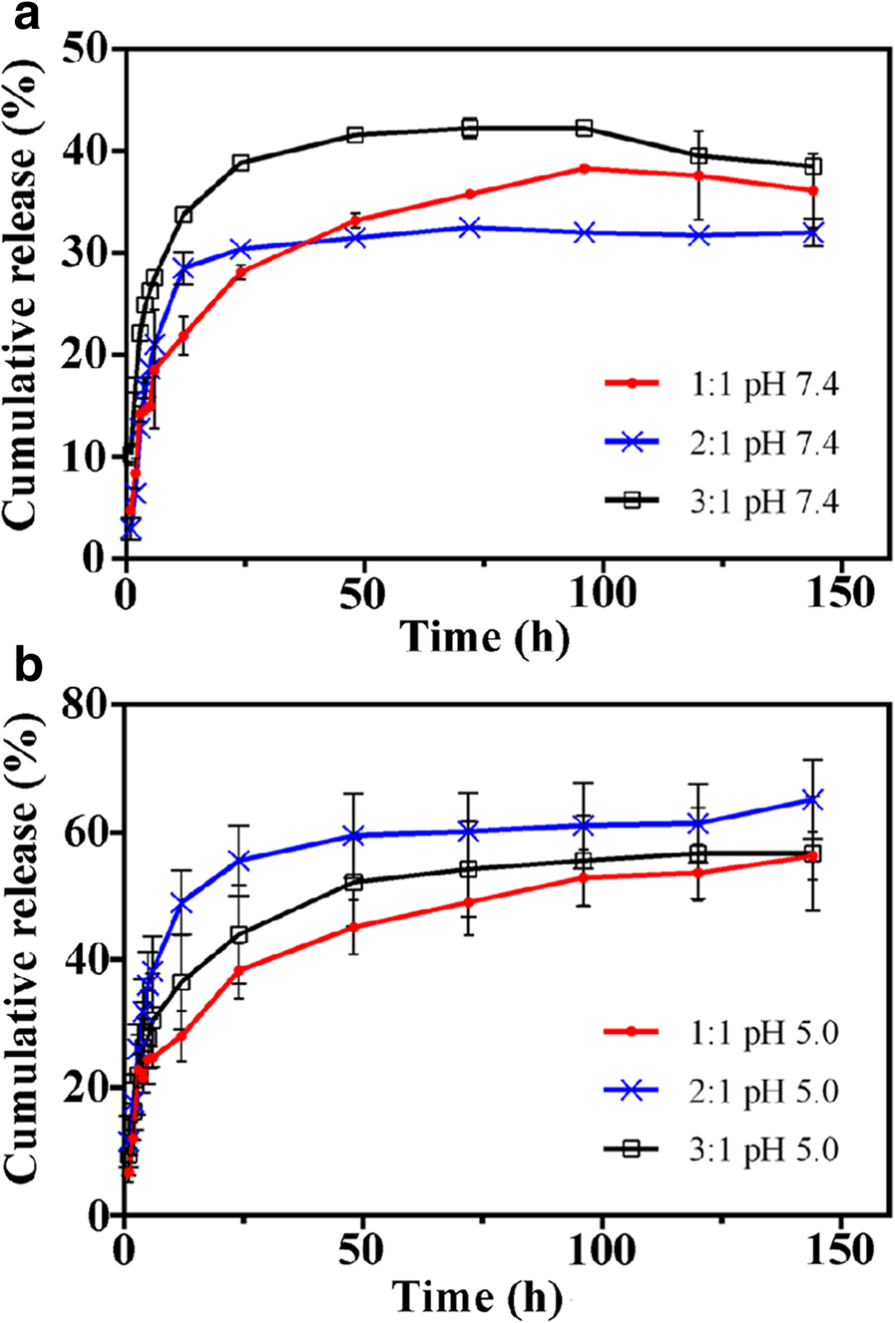
Release profiles of pH-responsive CA-PEI-pArg PMs at **a** pH 7.4 and **b** pH 5.0 (mean ± SEM, *n* = 3)

The release profiles of DOX-loaded PMs were studied at pH 7.4 and pH 5.0 (Fig. 4). PMs at all tested molar ratios demonstrated an initial burst release of loaded DOX in the first 12 h and achieved sustained release after 24 h. A similar burst release phenomenon has been reported by many researchers [38–40]. A small amount of DOX adsorbs to the hydrophilic region, instead of within hydrophobic core, due to the electrostatic interaction between DOX and the hydrophilic copolymers during micelle self-assembly. During the release process, these DOX molecules desorb from the micelles more readily than DOX loaded in the inner core, causing an initial burst release [39]. As shown in Fig. 4, DOX release from the PMs occurred much more rapidly at pH 5.0 than at pH 7.4. At pH 5.0 (Fig. 4b), PMs with a 2:1 molar ratio showed greater cumulative release (65%) after 6 days than the PMs with 1:1 or 3:1 molar ratios (56%). The increased release rate at acidic pH values was due to amine group protonation, which led to conformational changes and swelling of the micellar structure. At pH 7.4 (Fig. 4a), PMs with a 2:1 molar ratio released less DOX (32%) after 6 days than the PMs with 1:1 (36%) or 3:1 (38%) molar ratios. The rate of drug release from PMs with a 2:1 molar ratio plateaued after the first 48 h at pH 7.4, but gradually increased at pH 5.0, even after 5 days. This finding is relevant for the safety profile of DOX-loaded PMs, because the pH dependence of release facilitates secure circulation of the encapsulated drug in the blood and increases the release rate at the more acidic tumor interstitium. These findings (Fig. 4) were consistent with the DLS results (Fig. 3a, b). The PMs with a 2:1 molar ratio had the smallest size at pH 7.4 and released the least DOX, but their size drastically increased at pH 5.0 and they showed the greatest cumulative DOX release under these conditions. These findings show that a molar ratio of 2:1 is ideal for DOX delivery using CA-PEI-pArg PMs.

### f2 Similarity Test

The closeness of DOX release from CA-PEI-pArg PMs between pH 7.4 and 5.0 was investigated by fitting the release data to f2 equation (Table 1). Fitting of these data to f2 equation resulted in f2 values 73.11, 31.58, and 43.56, respectively, for CA-PEI-PArg PMs with molar ratios 1:1, 2:1, and 3:1. According to similarity factor f2, two profiles are considered to be alike when the f2 value is equal to 100. When the f2 value falls between 50 and 100, the difference between two profiles at each sampling time are less than or equal to 10% [41]. The results in Table 1 indicated that the differences in DOX release results at each sampling time for PMs with molar ratio 1:1 are less than 10%. On the other hand, the differences in DOX release rate from PMs with molar ratio 2:1 and 3:1 between pH 7.4 and 5.0 were recorded higher than 10%. Among all the PMs, molar ratio 2:1 recorded the lowest f2 values which is 31.58, indicated that the DOX release rate from PMs was very different in pH 5.0 as compared to pH 7.4.

**Table 1:** f2 similarity test for DOX release from CA-PEI-pArg PMs at pH 7.4 and 5.0

CA-PEI-pArg PMs	${\displaystyle \sum_{t=1}}n{\left({R}_t-{T}_t\right)}^2$	f2
1:1	141.69	73.11
2:1	7080.92	31.58
3:1	2339.80	43.56

### In Vitro Cellular Uptake

**Fig. 5:**
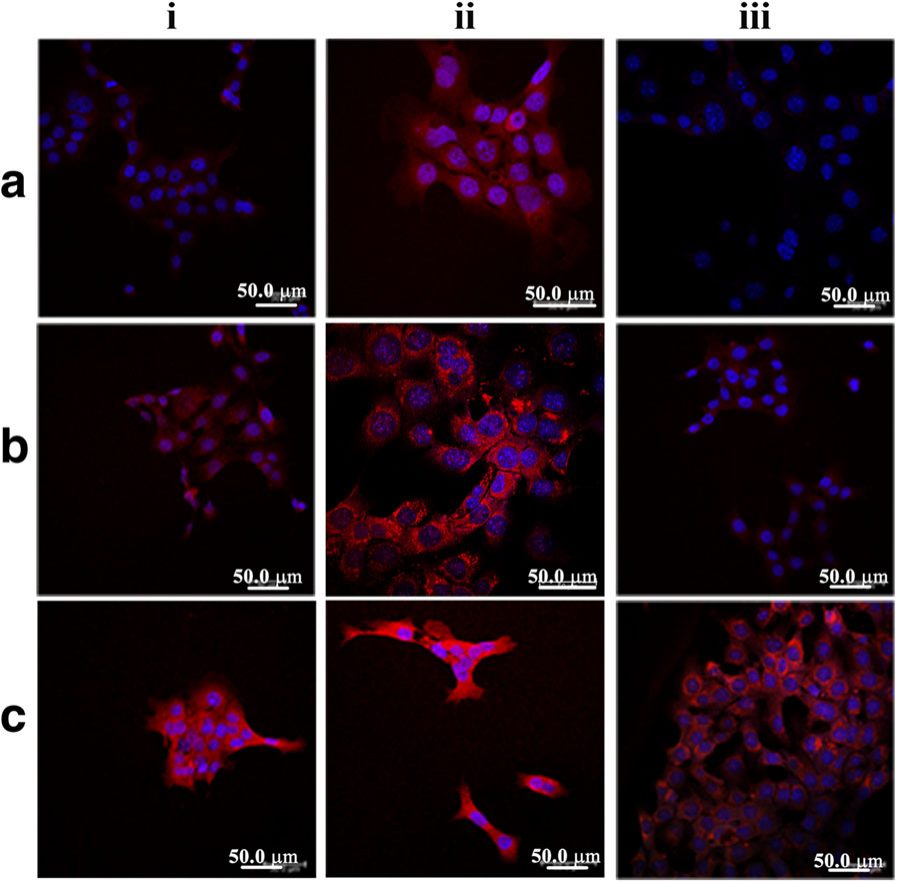
CLSM images of **a** free DOX solution, **b** DOX-loaded CA-PEI PMs, and **c** DOX-loaded CA-PEI-pArg PMs (CA:PEI molar ratio 2:1) in MCF-7 cells after incubation time of (*i*) 1 h, (*ii*) 4 h, and (*iii*) 24 h. *Blue* and *red* fluorescence indicate nuclei and DOX, respectively

**Fig. 6:**
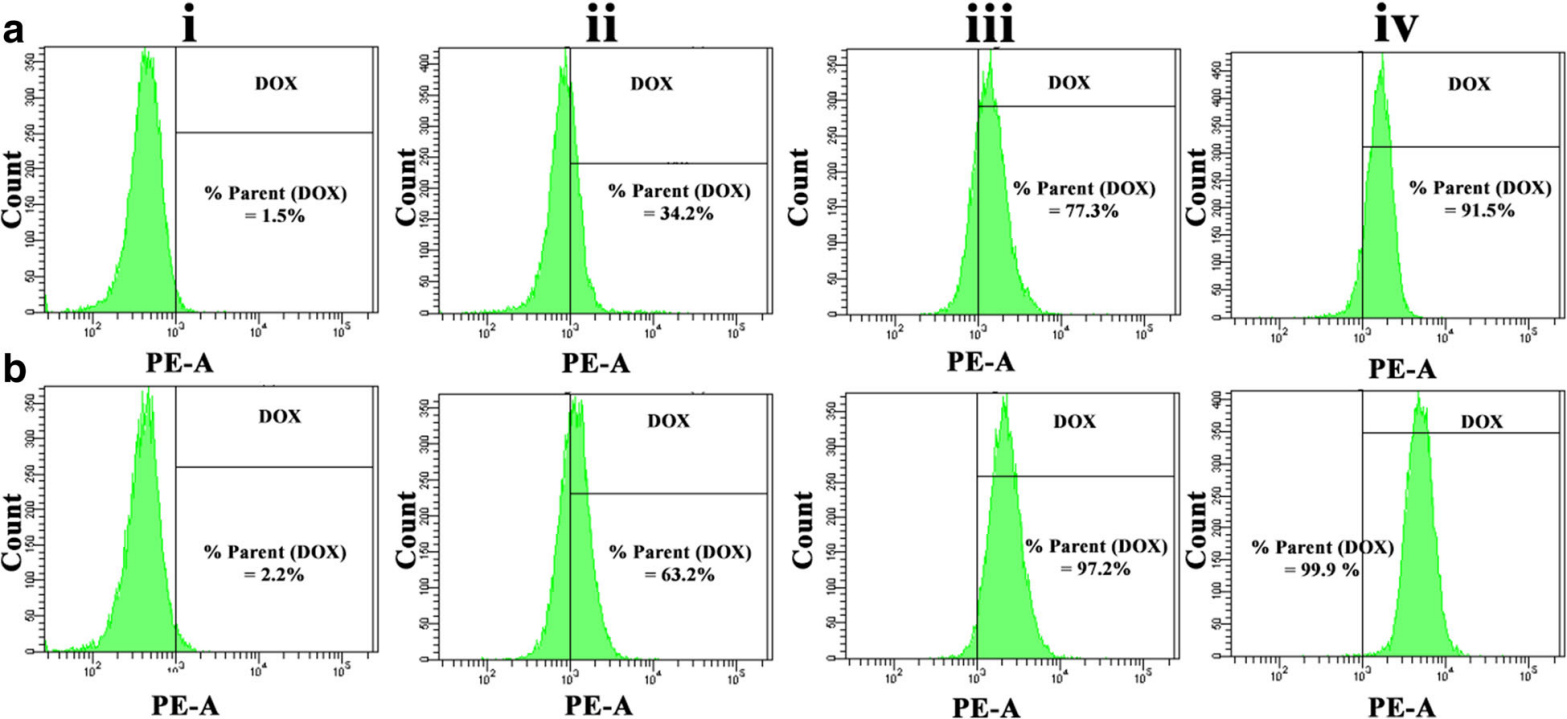
In vitro cellular uptake evaluation by flow cytometry on MCF-7 cells (*i*) without any treatment, (*ii*) treated with free DOX, (*iii*) treated with DOX-loaded CA-PEI PMs, and (*iv*) treated with DOX-loaded CA-PEI-pArg PMs after **a** 1-h and **b** 4-h incubation

To evaluate the cellular uptake efficiency, CLSM was used to localize DOX in MCF-7 cells. The CLSM images of MCF-7 cells after 1-, 4-, and 24-h incubations with free DOX solution, DOX-loaded CA-PEI PMs, or DOX-loaded CA-PEI-pArg PMs (10 μg/mL) are shown in Fig. 5. Weak red fluorescence was observed in MCF-7 cells incubated with free DOX for 1 h and 4 h (Fig. 5a (i, ii)). This red fluorescence was almost absent in MCF-7 cells incubated with free DOX for 24 h (Fig. 5a (iii)). This result suggested that the DOX has been removed from the cytoplasm and it could be due to the active removal of DOX by P-gp efflux pump. However, a greater red fluorescence intensity was observed in MCF-7 cells treated with DOX-loaded CA-PEI-pArg PMs (Fig. 5c) as compared to MCF-7 cells treated with DOX-loaded CA-PEI PMs (Fig. 5b) or free DOX, even after 24 h (Fig. 5c (iii)). The cellular uptake of DOX showed a time-dependent trend, with enhanced red fluorescence intensity observed as the incubation time increased from 1 h to 4 h (Fig. 5a–c (i, ii)]), in accordance to the flow cytometry results (Fig. 6). These results suggested that the use of DOX-loaded CA-PEI-pArg PMs enhanced the intracellular accumulation of DOX in MCF-7 cells, as compared to the use of the free DOX solution and DOX-loaded CA-PEI-pArg PMs. The results also showed that the cellular uptake of DOX has been improved with the presence of cell-penetrating pArg peptide in triblock PMs.

Cellular DOX uptake was also evaluated quantitatively by flow cytometry after 1 h and 4 h incubations. Intrinsic DOX fluorescence was detected in MCF-7 cells treated with DOX (Fig. 6 (ii-iv)), whereas no DOX fluorescence was detected in negative control cells (Fig. 6 (i)). As shown in Fig. 6, the fluorescence signal will appear in the right quadrant if MCF-7 cells had taken up the DOX, whereas the fluorescence signal will appear in the left quadrant if there is no DOX signal being detected in the MCF-7 cells. Result shows that the DOX fluorescence was detected in 34.2% (Fig. 6a (ii)) and 63.2% (Fig. 6b (ii)) of MCF-7 cells which were treated with free DOX solution for 1 h and 4 h, respectively. The percentage of MCF-7 cells detected with DOX fluorescence was recorded to be higher in MCF-7 cells treated with DOX-loaded CA-PEI-pArg PMs for 1 h (91.5%) (Fig. 6a (iv)) and 4 h (99.9%) (Fig. 6b (iv)), as compared to the MCF-7 cells treated with DOX-loaded CA-PEI PMs for 1 h (77.3%) (Fig. 6a (iii)) and 4 h (97.2%) (Fig. 6b (iii)). These findings suggest that the use of DOX-loaded CA-PEI-pArg PMs would achieve greater therapeutic efficacy due to enhanced cellular uptake into the cells. The presence of high amounts of DOX-loaded CA-PEI-pArg PMs in the MCF-7 cells could also be due to the small size of the PMs [42] or their cationic surface charge. The cationic surface charge of the PMs enhances contact with MCF-7 cells, and the use of small micelles facilitates internalization. In addition, the presence of pArg hydrophilic shell was expected to facilitate the cellular penetration of micelles across cell membrane [43].

### Cellular and Nuclear Morphology

**Fig. 7:**
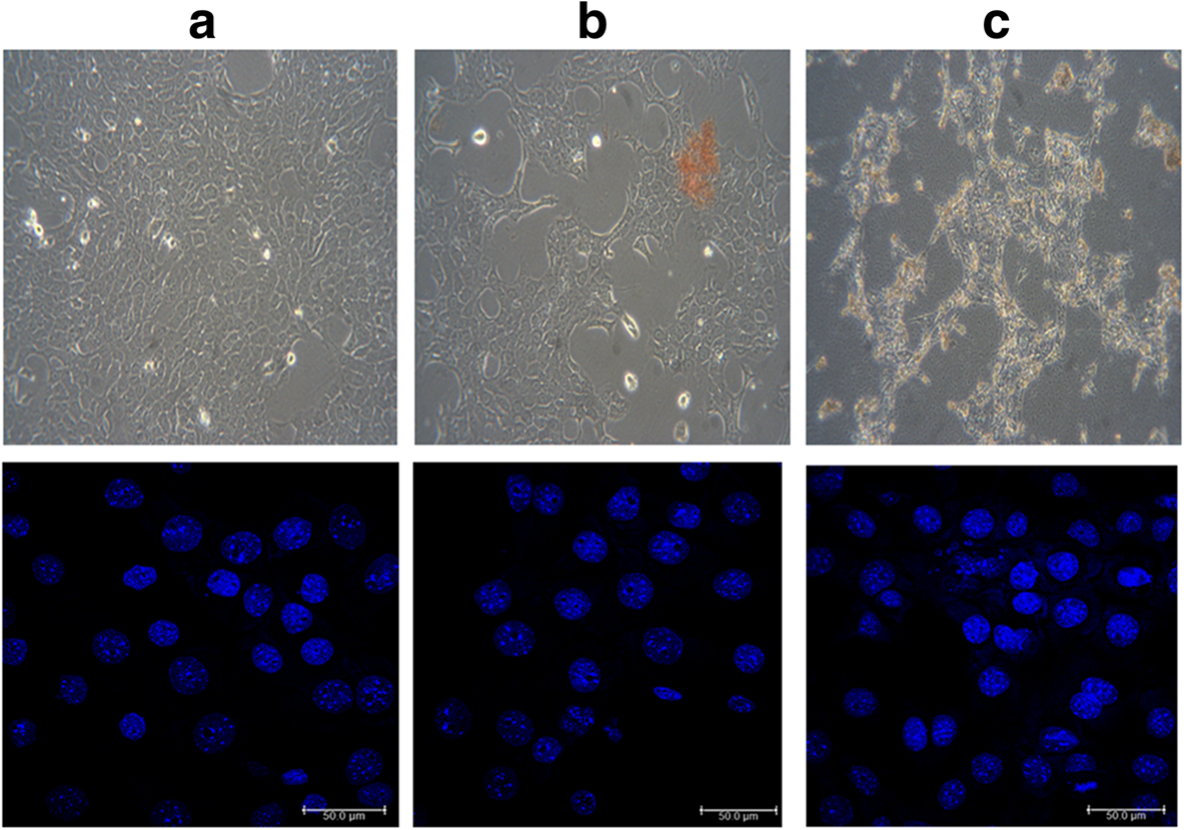
Cellular and nuclear morphologies of MCF-7 cells **a** untreated and after treatment with **b** free DOX solution and **c** DOX-loaded CA-PEI-pArg PMs (CA:PEI molar ratio 2:1) for 24 h

The morphologies of MCF-7 cells incubated with free DOX or DOX-loaded PMs (10 μg/mL) for 24 h were observed using CLSM and shown in Fig. 7. The cell nuclei were stained by Hoechst 33342. The cellular and nuclei morphology for MCF-7 cells exposed to free DOX solution (Fig. 7b) did not show prominent differences to the negative control cells (Fig. 7a). The nuclei morphology of MCF-7 cells exposed to DOX-loaded PMs (Fig. 7c) for 24 h did not show significant differences as compared to MCF-7 cells exposed to free DOX solution (Fig. 7b). However, there is a small number of nuclei undergone nuclear fragmentation in Fig. 7c but none were observed in Fig. 7b. In addition, significant morphological changes were observed in MCF-7 cells treated with DOX-loaded PMs for 24 h, including cell shrinkage, cell detachment, and nuclear fragmentation. These results indicated that MCF-7 cells exposed to DOX-loaded PMs showed signs of apoptosis more frequently than did cells exposed to the free DOX solution.

### Subcellular Localization

**Fig. 8:**
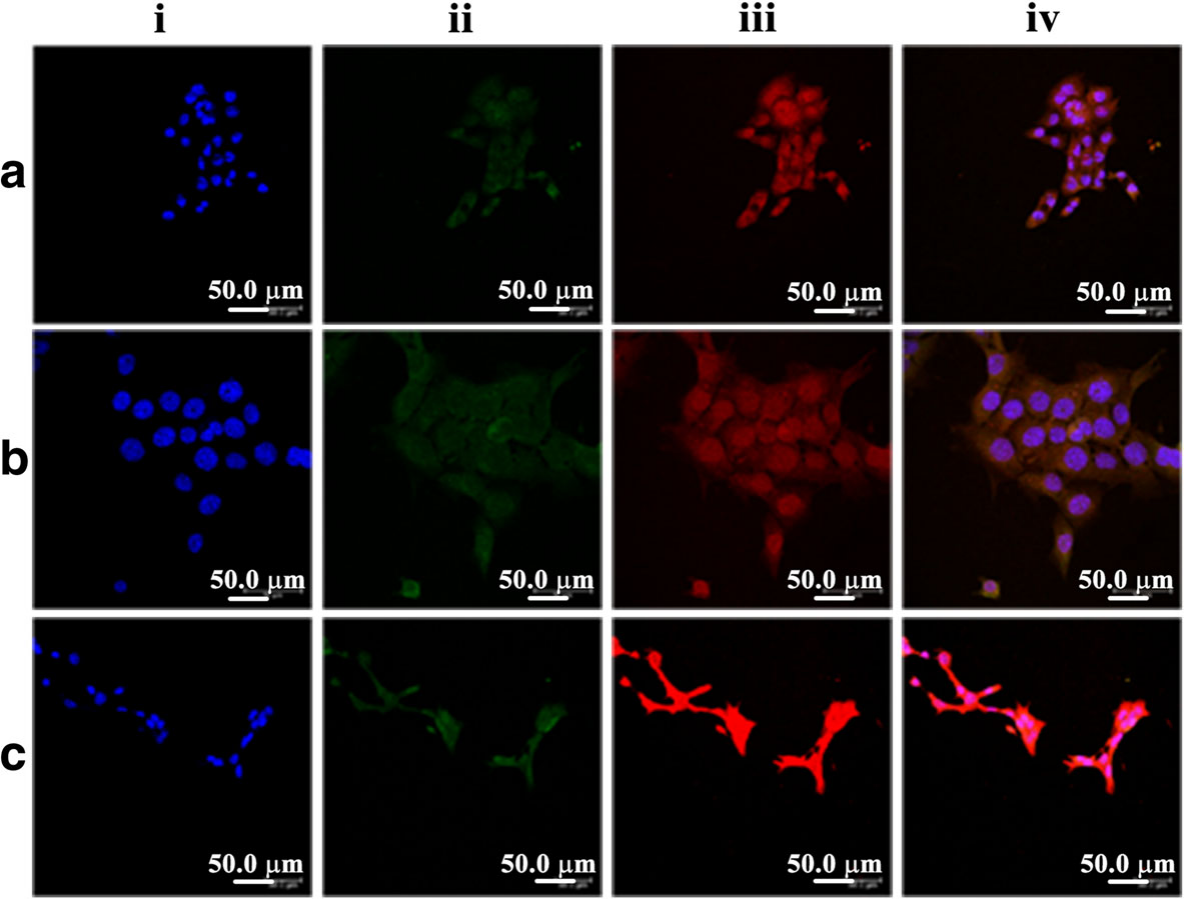
CLSM images of the subcellular localization of **a** free DOX solution, **b** DOX-loaded CA-PEI PMs, and **c** DOX-loaded CA-PEI-pArg PMs (CA:PEI molar ratio 2:1) in MCF-7 cells after 4-h incubation: (*i*) cell nuclei (*blue*); (*ii*) lysosomes (*green*); (*iii*) DOX (*red*); and (*iv*) overlay of all images

The localization of DOX to the lysosomal compartment in MCF-7 cells was also observed by CLSM (Fig. 8). The nuclei were stained blue by using Hoechst 33342; lysosomes were stained green by using a Lysotracker Green; and DOX were appeared as red owing to its intrinsic fluorescence. Colocalization of DOX with lysosomes appeared as yellow signal, while non-colocalization appeared as red signal. MCF-7 cells treated with free DOX solution (Fig. 8a (iv)) showed yellow compartments, suggesting that DOX was trapped in the lysosomes of MCF-7 cells. The red fluorescence signal observed in these MCF-7 cells was very weak. According to the literature, some of the hydrophobic drugs such as DOX, daunorubicin, mitoxantrone, imidazoacridinones, and sunitinib were reported to accumulate in the lysosomes instead of cytoplasm [44–48]. At physiological pH, the hydrophobic amine drugs will diffuse across the cell membrane via passive diffusion. These drugs will undergo protonation upon entry into the lysosomes or late endosomes with acidic pH and eventually entrapped in the lysosome [49]. As a result, the hydrophobic amine drugs will be sequestered away and prominently reduced the accumulation of these drugs in the nucleus [50]. In contrast, MCF-7 cells treated with DOX-loaded CA-PEI-pArg PMs showed a higher intensity of red fluorescence (Fig. 8c (iv)) as compared to those incubated with DOX-loaded CA-PEI PMs (Fig. 8b (iv)) or free DOX solution. These results suggested that the use of DOX-loaded CA-PEI-pArg PMs facilitates the escape of DOX from the lysosomal compartment, allowing it to reach the cytoplasmic and nuclear regions of the MCF-7 cells. In addition, the results of this study are in consistence with the cellular uptake study as shown in Fig. 5. The MCF-7 cells exposed to DOX-loaded CA-PEI-pArg PMs (Fig. 8c (iii)) showed stronger DOX fluorescence signal than those exposed to free DOX solution (Fig. 8a (iii)) or DOX-loaded CA-PEI PMs (Fig. 8b (iii)), indicated improved cellular uptake of DOX-loaded PMs into the MCF-7 cells. Apart from the improved cellular uptake efficiency, the incorporation of pArg into CA-PEI-pArg copolymer was also shown to improve the escape of DOX from lysosome. This could be a contribution from the protonation of amine groups in pArg under acidic environment, which would lead to osmolarity changes in the lysosome and eventually destabilize the lysosome structure.

### In Vitro Cytotoxicity of PM

**Fig. 9:**
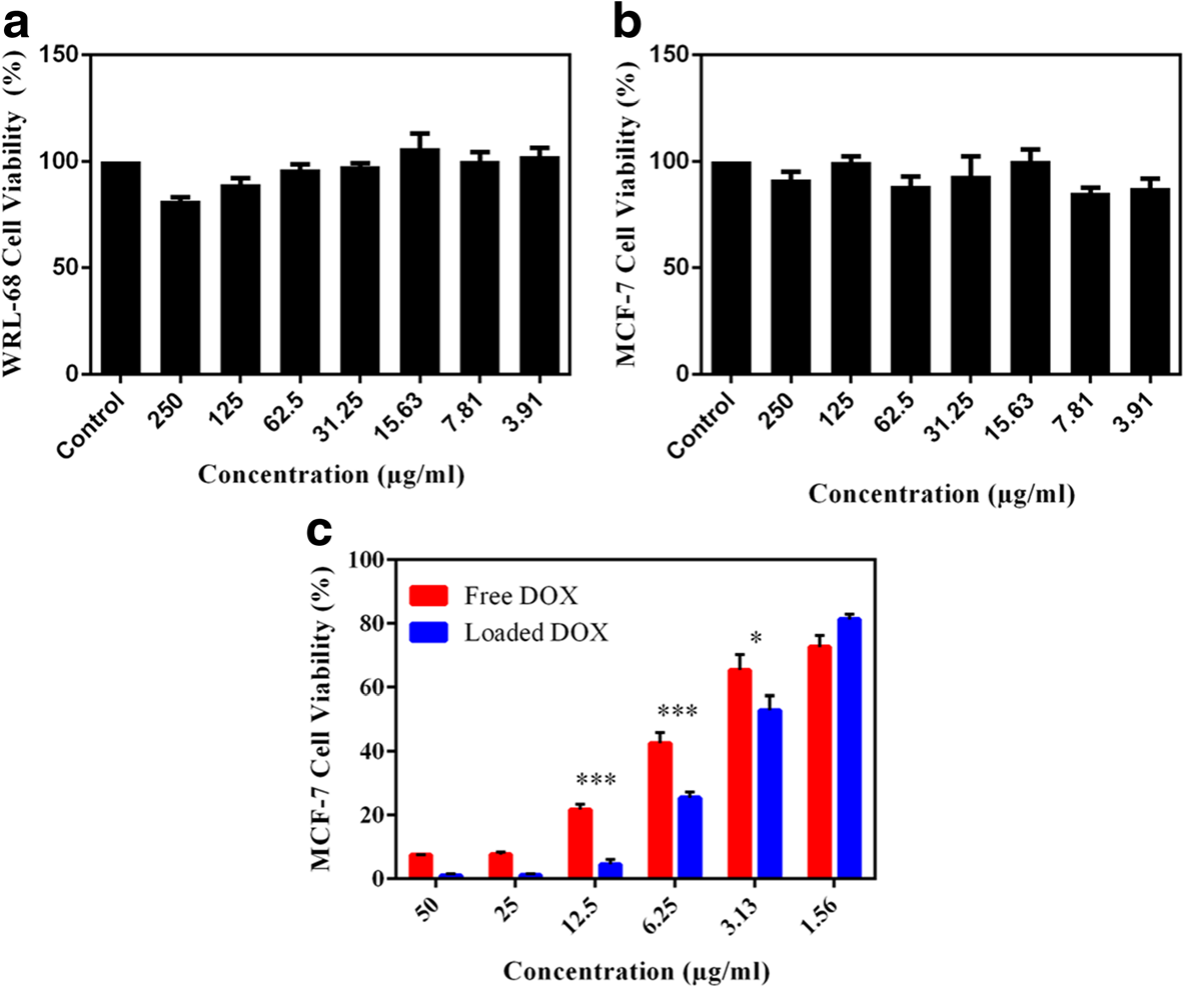
**a** Cytotoxicity of blank CA-PEI-pArg PMs (CA:PEI molar ratio 2:1) in WRL-68 cells after 24 h. **b** Cytotoxicity of blank CA-PEI-pArg PMs in MCF-7 cells after 24 h. **c** Cytotoxicity of DOX-loaded CA-PEI-pArg PMs (CA:PEI molar ratio 2:1) in MCF-7 cells after 24 h (mean ± SEM, *n* = 3; *P ≤ 0.05, ***P ≤ 0.001)

The safety profile of blank CA-PEI-pArg PMs in WRL-68 cells and MCF-7 cells were evaluated by MTT assay (Fig. 9a, b). More than 90% of cells were viable at all tested concentrations, suggesting that the blank micelles were not toxic to WRL-68 cells and MCF-7 cells at concentrations of ≤250 μg/mL. These results indicated that the blank PMs did not cause cytotoxic or inhibitory effects on human hepatic cells (WRL-68) or breast cancer cells (MCF-7).

The effect of DOX-loaded CA-PEI-pArg PMs on cell viability was examined using MCF-7 cells. MCF-7 cells were treated with DOX-loaded PMs or free DOX solution (1.562–50 μg/mL). After 24-h incubations, cell viability was assessed by the MTT assay (Fig. 9c). The inhibitory effect of DOX-loaded PMs (3.13–50 μg/mL) on MCF-7 cells was greater than that of the free DOX solution. The cell viability of MCF-7 cells treated with DOX-loaded PMs (3.13 – 12.5 μg/mL) were significantly lower than MCF-7 cells treated with free DOX solution. These MTT results were consistent with the results of the cellular uptake experiments, confirming that the PM-associated increased penetration of DOX through the cell membrane resulted in a greater inhibitory effect. These findings also suggested that the proton sponge effect of PEI successfully triggered endosome escape and release of the drug into the cytoplasm. Accumulation of DOX in the cytoplasm could damage the nucleus and enhance the cytotoxicity of DOX. However, the cytotoxicity effect of DOX-loaded PMs was slightly lower than free DOX at concentration of 1.56 μg/mL; this may reflect over-dilution of DOX-loaded PMs during the preparation of the formulations using serial dilution. No micelle formation occurs when the concentration of block copolymers is lower than the CMC. Therefore, there is no significant difference between the cytotoxicity effect caused by loaded DOX and free DOX at concentration 1.56 μg/mL.

## Conclusions

In this study, a pH-responsive drug delivery system was developed to evaluate its efficacy for delivering DOX to human breast cancer MCF-7 cells. FTIR and ^1^H NMR confirmed the successful synthesis of triblock CA-PEI-pArg copolymers. Inclusion of a pH-sensitive block (PEI) conferred pH-dependent changes in particle size, drug release profiles, and drug release kinetics. Inclusion of pArg as a hydrophilic shell increased cellular uptake and enhanced the cytotoxicity of DOX-loaded PMs to MCF-7 cells. This study indicates that controlled release of anticancer drugs at acidic pH values can be achieved using pH-responsive PMs. The combination of pH-dependent drug release, a cell-penetrating peptide, and proton sponge-triggered endosomal escape produced a robust pH-responsive drug delivery system with high efficacy, warranting further investigation.
